# Evaluation of Wound Closure Activity of *Nigella sativa*, *Melastoma malabathricum*, *Pluchea indica*, and *Piper sarmentosum* Extracts on Scratched Monolayer of Human Gingival Fibroblasts

**DOI:** 10.1155/2014/190342

**Published:** 2014-10-13

**Authors:** Mas Rizal Ab Rahman, Fathilah Abdul Razak, Marina Mohd Bakri

**Affiliations:** Department of Oral Biology and Biomedical Sciences, Faculty of Dentistry, University of Malaya, 50603 Kuala Lumpur, Malaysia

## Abstract

*Nigella sativa*, *Melastoma malabathricum*, *Pluchea indica*, and *Piper sarmentosum* are common Asian traditional medicines to treat minor wounds. This study aimed to investigate the *in vitro* wound healing properties of aqueous extracts of these plants using human gingival fibroblast (HGF) monolayer as study model. DPPH scavenging activity of the extracts was evaluated and effect on HGF proliferation was determined. Their effect on HGF's function to synthesize collagen was indicated by the level of hydroxyproline produced and effect on wound healing activity was assessed using an *in vitro* scratch assay. The influence of the extracts on expression of bFGF and TGF-*β* was also determined. Results revealed all four extracts to exhibit low free radical scavenging activity. The extract from *N. sativa* (NSSE) compared to the others showed favourable enhancement of HGF proliferation with EC_50_ of 22.67 ± 3.06 *µ*g/mL (*P* < 0.05) with accelerated wound closure activity despite its nonsignificant effect on collagen synthesis. In addition to the elevated level of bFGF by up to 15% at 100 *µ*g/mL of NSSE, a slightly better effect was observed on the expression of TGF-*β*. NSSE thus showed that promising wound healing properties and data obtained may contribute towards validation of its traditional use for the healing of oral wounds.

## 1. Introduction

Due to the widespread belief that “green medicine” is safe, the use of plant products is perceived as effective, better tolerated by patients, and less expensive [1, 2]. Preparations from plants have been used since ancient time to accelerate the process of wound healing. The efficacy of these medicines relies exclusively on practical experience and observation passed on verbally from one generation to the next with little supporting documentation. The search for active compounds from natural resources has been actively pursued as it is necessary to understand the contemporary relevance of this traditional knowledge and wherever possible to elucidate the mechanism involved.

Wounds are physical injuries that resulted in an opening or break of the skin [3]. Wounds to the oral mucosa may result from physical, chemical, and mechanical activities or traumas [4]. Despite reports on the accelerated healing process and scarless wounds in the oral cavity mentioned by Glim et al. [5], in contrast to wounds on the skin, curing oral-associated wound is a challenge due to the continuous flow of saliva and presence of microorganisms that tend to interrupt and slows down the healing process. Wound healing consists of an orderly progression of events that reestablish the integrity of the damaged tissue: inflammatory, proliferation, and remodeling stages [6]. The different phases of the wound healing process overlap and ideally at least two different processes should be affected by a plant-based remedy before it is said to have wound healing properties [7]. Many plants in the crude form or identified active-components have been investigated for their healing effects of skin wounds. Less effort, however, was focused on the healing of wounds occurring in the oral cavity.

According to Roy et al. [8],* Nigella sativa*,* Pluchea indica*,* Melastoma malabathricum,* and* Chromolaena odorata* are some of the plants that have been widely used in local practices to heal minor wounds. Research has shown that the extract from* N. sativa* can heal burn-related skin wounds in rat model [9] and topical application of oil prepared from its seeds can accelerate wound healing [10]. In relation to the oral environment,* N. sativa* seeds have been reported to prevent the formation of dental plaques and caries [11]. Topical application of poultice prepared from leaves of* P. indica* and* M. malabathricum* is also commonly practiced by the local people to treat minor wounds and this traditional medication was also reported by other researches within the region [12, 13].* C. odorata* is another medicinal plant whose effectiveness in healing soft tissue and burn wounds was reported by researchers from Vietnam [14].

This study aimed to investigate the healing activity of aqueous extracts of* N. sativa*,* M. malabathricum*,* P. indica,* and* P. sarmentosum* on wounds created on monolayer of oral fibroblasts. Fibroblasts are cells responsible for the generation of collagen fibers in the connective tissues of the oral mucosa and thus represented a good target for the study.

## 2. Material and Methods

### 2.1. Materials

#### 2.1.1. *N. sativa*

*N. sativa* is locally known as* Abbatus sauda* or simply as* black seeds*. In the South Asia,* N. sativa* is known as Kalonji, and in the English literature, it is known as black cumin [15]. Listed under the family of* Ranunculaceae,* the popular medicinal usage of this plant in the local scenario has been based on the Muslims' believe of its healing ability. The various biological activities of* N. sativa* are mentioned in the Quran. The black seeds are easily available in the market as it is also being used as cooking condiment. Authentication of the seeds was made by a botanist at the Botanical Garden Herbarium, University of Malaya. Reference for voucher specimen is under preparation.

#### 2.1.2. *M. malabathricum*

*Melastoma malabathricum* falls under the family of* Melastomataceae*. It is known as* Senduduk* by the local people of Malaysia and is considered a weed in Malaysian plantation crops as it grows wild in abandoned wastelands where sunshine is abundant. Senduduk consists of three varieties, having large, medium, and small size flowers made up of either dark purple-magenta petals, light pink-magenta petals, or white petals (the rare variety). The white flower of* M. malabathricum* is reported to have miraculous healing properties [16]. The type used in the study is the purple-flower species. The plants are evergreen and flower throughout the year [17]. Authentication of the seeds was made by a botanist at the Botanical Garden Herbarium, University of Malaya (voucher specimen no. KLU47673).

#### 2.1.3. *P. indica*

*Pluchea* is a genus of flowering plant in the* Asteraceae* family native to tropical and warm temperate areas. In general, the plants of* Pluchea* genus have been traditionally used as astringent, antipyretic, anti-inflammatory, hepatoprotective, diaphoretic in fevers, smooth muscle relaxant, nerve tonics, and laxatives and for the treatment of dysentery, lumbago, leucorrhoea, dysuria, haemorrhoids, gangrenous ulcer, and disorders causing cachexia [18]. Members of this genus have many names and might be known as camphorweeds, plucheas, or less uniquely “fleabanes.” Pharmacological studies demonstrated anti-inflammatory and antioxidant activities of different* Pluchea sp*. [19] which is believed to play an important role in the early stage of wound healing. Authentication of* Pluchea indica was* made by a botanist at the Botanical Garden Herbarium, University of Malaya (voucher specimen no. KLU39445).

#### 2.1.4. *P. sarmentosum*

Native to Malaysia and Indonesia,* Piper sarmentosum*, locally known as “kaduk,” is under the family of* Piperaceae*. Known as “Cha-plu” in Thailand,* P. sarmentosum* grows easily both in partially or exposed sites and on a wide range of soils. It is widely distributed in the tropical and subtropical region of the world and is often used as food flavouring agents and traditional medicines [17]. In Malaysia, they are also eaten raw as* ulam* and the leaves are boiled in water and taken to relieve fever in malaria and treat coughs, flu, and rheumatism. They are also chewed with ginger to relieve tooth pain. In the Malay and Indonesian population, the leaves and roots of this plant are used for the treatment of toothache, fungoid dermatitis on the feet, coughing asthma, and pleurisy [20]. Authentication of* Piper sarmentosum* was made by a botanist at the Botanical Garden Herbarium, University of Malaya (voucher specimen no. KLU47820).

### 2.2. Methods

#### 2.2.1. Preparation of Aqueous Plant Extracts

The protocol used for extraction in this work reflects the method of preparation used by traditional healers in Malaysia. Seeds of* N. sativa* and leaves of* M. malabathricum*,* P. indica,* and* P. sarmentosum* were cleaned and 100 g of each specimen was weighed, ground, and placed in separate conical flasks containing 1000 mL of distilled water. Decoction of each plant was prepared overnight [21] and debris was removed by passing the extract through muslin cloth followed by a filter paper (Whatman #1). The concentrates were dispensed into several glass flasks and prepared to be freeze-dried (EYELA FDU 1200) overnight. The dry extracts were then appropriately weighed for use in the experiments.

#### 2.2.2. DPPH Scavenging Assay (2,2-Diphenyl-1-picrylhydrazyl Free Radical Test)

*(1) Samples Preparation*. An aliquot of ethanol containing solution of different concentration (0, 50, 100, 200, 400, 600, 800, and 1000 *µ*g/mL) plants extracts was prepared. To compare the activity of plants extracts, ascorbic acid was used as a positive control. A concentration of 0, 1.25, 2.5, 5.0, 7.5, 10, 12.5, and 15 *µ*g/mL of ascorbic acid was prepared.

*(2) DPPH Assay*. The effect of the antioxidant on DPPH radical was estimated according to the procedure described by Nordin [22]. All the spectrophotometric data were acquired using an UV-1800 Shimadzu in a 10 mm quartz cuvette. 2.5 mL of each concentration of extracts and ascorbic acid prepared were added into 1 mL of DPPH and they were left in dark for 30 min. After 30 min, the solution was transferred into quartz vial and reading was taken at 518 nm. Ethanol was used to zero the spectrophotometer. The absorbance of DPPH radical without any extract was considered as control. IC_50_ value was obtained by setting a graph percentage of inhibition against sample concentration. The percentage of inhibition was calculated as follows:

(1) % of DPPH's inhibition:Absorbance(control)−Absorbance(sample)Absorbance(control)×100.

Finally the percentage of inhibition against standard concentration was plotted in an exponential regression to obtain the amount of extract needed to decrease the initial DPPH concentration by 50% (IC_50_).

#### 2.2.3. Determination of the Effect of Extracts on HGF Proliferation

*(1) Preparation of HGF Cell Line*. Fibroblast cell line was developed from an explant of gingival tissues scraped off an extracted tooth of a patient during an extraction procedure at the Oral Surgery Clinic, Faculty of Dentistry, University of Malaya [ethic approval of MEC : DF OB1002/0039(P)]. Stimulation of fibroblast growth was performed according to methods of Adetutu et al., [23] with slight modification. Fibroblast growth was closely monitored to obtain the best condition for use in the study. Cells of passages 4 to 9 upon reaching 90% confluent were used in the experiments. Dulbecco's Modified Eagle Medium (DMEM) containing 10% fetal bovine serum (FBS), 2% penicillin/streptomycin, and 1% amphotericin B was used to revive the fibroblasts. A cell suspension at a concentration of 3 × 10^3^ cells/mL was then prepared for use in the assay.

*(2) Assay for Cell Proliferative Activity*. HGF cell line was dispensed in a 96-well plate at 3 × 10^3^ cells/well. Once ready, the plate was placed in a humidified incubator at 37°C and 5% CO_2_ atmosphere to allow for formation of fibroblast monolayer in each well. After 48 h, the plate was taken out and the growth media were removed from the newly formed fibroblasts monolayer and replaced with a new DMEM supplemented with a lower percentage of FBS (0.3%) to meet basic growth requirement of the fibroblasts. This was done to create a minimal growth condition for the fibroblasts [23]. Plant extracts at concentrations ranging from 1 to 100 *µ*g/mL were then added to the monolayers. DMEM/0.3% FBS and DMEM/10% FBS in the absence of the extracts were used as the negative and positive control for the experiment, respectively. Once ready, the plate was incubated for another 48 h to allow for reaction to take place. Following incubation, the plate was taken out and the medium was discarded. The viability of the extract-treated HGF was determined using the neutral red staining procedure [24]. The test was carried out in triplicate and repeated three times for standardization and reproducibility. Absorbance of the colour reaction was read at a wavelength of 540 nm and was presented as mean percentage increase ± standard deviation of the mean (SD). Percentage increase of HGF population was calculated as

(2) Percentage increase (%)=Absorbance(sample)−Absorbance(negative control)Absorbance(negative control)×100.

#### 2.2.4. Determination of the Effect of Extracts on Wound Closure Activity

The scratch assay by Fronza et al. [25] was employed to assess the effect of the extracts on the wound closure ability of HGF. Monolayers of HGF were allowed to form in a 6-well plate containing an enriched medium of DMEM/10% FBS. Upon nearly confluent, the medium was discarded and replaced by a basic medium of DMEM/0.3% FBS to minimally maintain the growth of HGF. After 24 h of incubation, the plate was taken out and artificial wounds were created in the monolayers by making a linear scratch in the centre of each well using the tip of a sterile 1000 *µ*L plastic pipette tip. Any cellular debris created from the scratch was removed by gently washing the wells with phosphate buffered saline (PBS).

Once ready, the scratched wounds were divided into three groups in triplicate. In the first group, DMEM/0.3% FBS was added to represent as negative control. In the second group, 10 ng/mL of basic fibroblast growth factor (bFGF) which is a growth enhancer was added to represent as positive control. In the third group, 25 *µ*g/mL of the extracts was instead added to represent the test group. All plates were then incubated at 37°C in a humidified incubator with 5% CO_2_ atmosphere. The plate was periodically taken out at varying stages of 0, 6, 18, 30, 42, and 54 h of incubation to monitor the closure of the scratched wounds. Micrographs to record the wound closure activity at each stage were captured under an inverted microscope (Olympus, CK40).

#### 2.2.5. Determination of the Effect of Extracts on Collagen Synthesis

*(1) Preparation for Collagen Synthesis*. The assay was performed following the protocol of Cilli et al. [26] with slight modification. HGF were seeded into 4 columns of a 96-well plate at a density of 1 × 10^3^ cells/well in DMEM/10% FBS and incubated at 37°C in a humidified incubator of 5% CO_2_ atmosphere. After 24 h, the plate was taken out and the medium was discarded. Basic media of DMEM/0.3% FBS were added to the first column of wells to represent as negative control for the test while those in the second column were added with an enriched medium of DMEM/10% FBS to represent as positive control. In the third column, 25 *µ*g/mL of extract in DMEM/0.3% FBS was added to represent as test samples while, in the fourth column, 25 *µ*g/mL of allantoin in DMEM/0.3% FBS was added for comparative purposes. Allantoin is a common epithelial enhancer used in skin care products. The plate containing the treated-HGF was then incubated for 72 h to allow time for collagen synthesis to take place. Following incubation, the medium in each well containing collagen synthesised by the treated-HGF was carefully pipetted out into clean vials. A volume of 0.5 mL of 6 N HCL was added to hydrolyse the collagen. The vials were then autoclaved for 20 min at 120°C and once cooled the concentration of hydroxyproline in the hydrolysed medium which corresponded to the amount of collagen synthesised by HGF in each vials was determined and analysed according to the following procedure.

*(2) Hydroxyproline Analysis*. The concentration of hydroxyproline was determined using a kit (Biovision, USA). Briefly, 10 *µ*L of the hydrolysed medium was mixed with 100 *µ*L of chloroamines T solution. Following incubation for 5 min at room temperature, 100 *µ*L of dimethylaminoborane (DMAB) solution was added and incubation was continued for another 90 min at 60°C. The plate was then removed from the incubator and the absorbance was read spectrophotometrically using a plate reader (uQuant, USA) at a wavelength of 560 nm. These readings were then compared to a standard curve to determine the content of hydroxyproline. A series of hydroxyproline at concentrations ranging from 0 to 10 *µ*g/mL was used in the preparation of the standard curve. Each sample was analysed in sextuple (*n* = 6).

#### 2.2.6. Determination of the Effect of Extracts on the Expression of Basic Fibroblast Growth Factor (bFGF) and Transforming Growth Factor-Beta (TGF-*β*)

*(1) Preparation of HGF Suspension in Serum-Free Medium*. HGF was cultured in DMEM/10% FBS to confluent in a 25 cm^2^ culture flask. The cells were then detached by the addition of Accutase. The detached cells were resuspended in a serum-free medium containing 1% of Glutamax. It was found earlier in a pilot study that glutamax is required to maintain the survival of HGF in a serum-free medium. A suspension of the detached cells at a density of 6 × 10^4^ cells/mL was then prepared and seeded into 6 wells of a 24-well plate. Every two wells were categorized as Group 1, Group 2, and Group 3. The plate was incubated for 24 h at 37°C in 5% CO_2_ atmosphere.

*(2) Treatment of HGF*. Following incubation, the medium was discarded and a single wash was performed using PBS to remove any dead or nonadherent cells. A volume of 1.5 mL of serum-free medium (with 1% glutamax) containing the extracts at two different concentrations, 25 *µ*g/mL and 100 *µ*g/mL, was added to wells of Group 1 and Group 2, respectively. Wells in Group 3 contained only 1.5 mL of serum-free medium (with 1% glutamax) to represent as a negative control. The culture plate was further incubated for 48 h.

*(3) Assessment of bFGF*. After 48 h, the supernatant from wells of Group 1, Group 2, and Group 3 was pooled and the concentration of bFGF in each group was determined using solid phase enzyme-linked immunosorbent assay (ELISA). To determine the concentration of bFGF, a FGF2 (Human) ELISA kit (Abnova, USA) with a detection limit of 78 pg/mL was used. The production of bFGF by HGF was detected by a colour change which was read using an ELISA reader at a wavelength of 450 nm. A standard curve was used to determine the content of bFGF. A series of bFGF at concentrations ranging from 0 to 8000 pg/mL was plotted to produce the standard curve. Each concentration and each control were determined in triplicate (*n* = 3).

*(4) Assessment of TGF*-*β*. After 48 h, the supernatant from wells within Group 1, Group 2, and Group 3 was pooled and the concentration of TGF-*β* in each group was determined using solid phase enzyme-linked immunosorbent assay (ELISA). To determine the concentration of TGF-*β*, a TGF-*β* (Human) ELISA kit (Abnova, USA) with a detection limit of 78 pg/mL was used. The production of TGF-*β* by HGF was detected by a colour change which was read using an ELISA reader at a wavelength of 450 nm. A standard curve was used to determine the content of TGF-*β*. A series of TGF-*β* at concentrations ranging from 0 to 8000 pg/mL was plotted to produce the standard curve. Each concentration and each control were determined in triplicate (*n* = 3).

#### 2.2.7. Statistical Analysis

Data obtained from the experiments were compared to those of the negative and positive controls and the results were statistically analyzed using one-way ANOVA parametric test SPSS version 11.5. Results were considered significant at *P* < 0.05.

## 3. Results

In comparison to ascorbic acid, the free radical scavenging activity of all four plants extract was insignificant (Table 1). Although the activity exhibited by* P. indica* was the highest among the four extracts, it was still 27-fold less than that of ascorbic acid.

**Figure 1:**
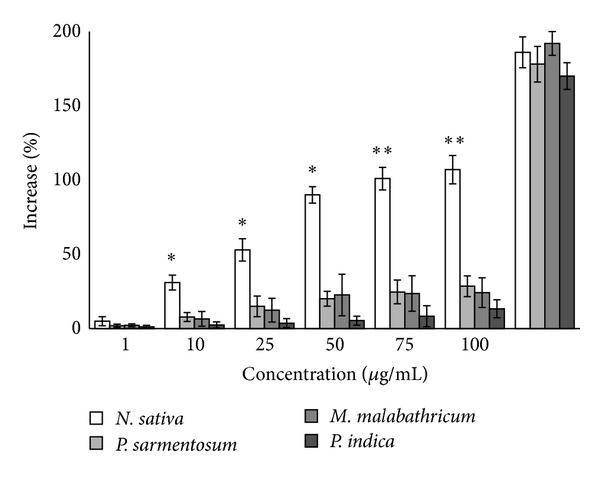
The proliferative effect of four plants extracts on HGF as indicated by the increase in percentage of HGF population following exposure to the extracts. The rate of cell proliferation of NSSE-treated cell was linear at concentrations below 50 *µ*g/mL but slowed down at higher concentrations above 50 *µ*g/mL. The values plotted were the mean of triplicate tests (*n* = 3). *P* values at *P* < 0.05 were indicated by (∗) and at *P* < 0.01 by (∗∗).

Based on results obtained from the fibroblast stimulation assay, a favourable increase in HGF population was observed when the cells were treated with NSSE and this proliferative activity was found to be concentration-dependent. 31% increase in HGF count was recorded at 10 *μ*g/mL and a maximum 90% increase was obtained at 50 *µ*g/mL of NSSE (*P* < 0.05). The effective concentration producing 50% HGF proliferation (EC_50_) was determined at 22.67 ± 3.06 *µ*g/mL. Proliferation of HGF however slowed down at concentrations higher than 50 *μ*g/mL (Figure 1). The leave extracts (100 *μ*g/mL) of* M. malabathricum*,* P. indica*, and* P. sarmentosum* exhibited very minor HGF proliferative activity at 24.2%, 13.4%, and 28.6%. Considering the minor proliferative activity, extracts of* M. malabathricum*,* P. indica*, and* P. sarmentosum* were excluded from further wound closure assessment.

**Figure 2:**
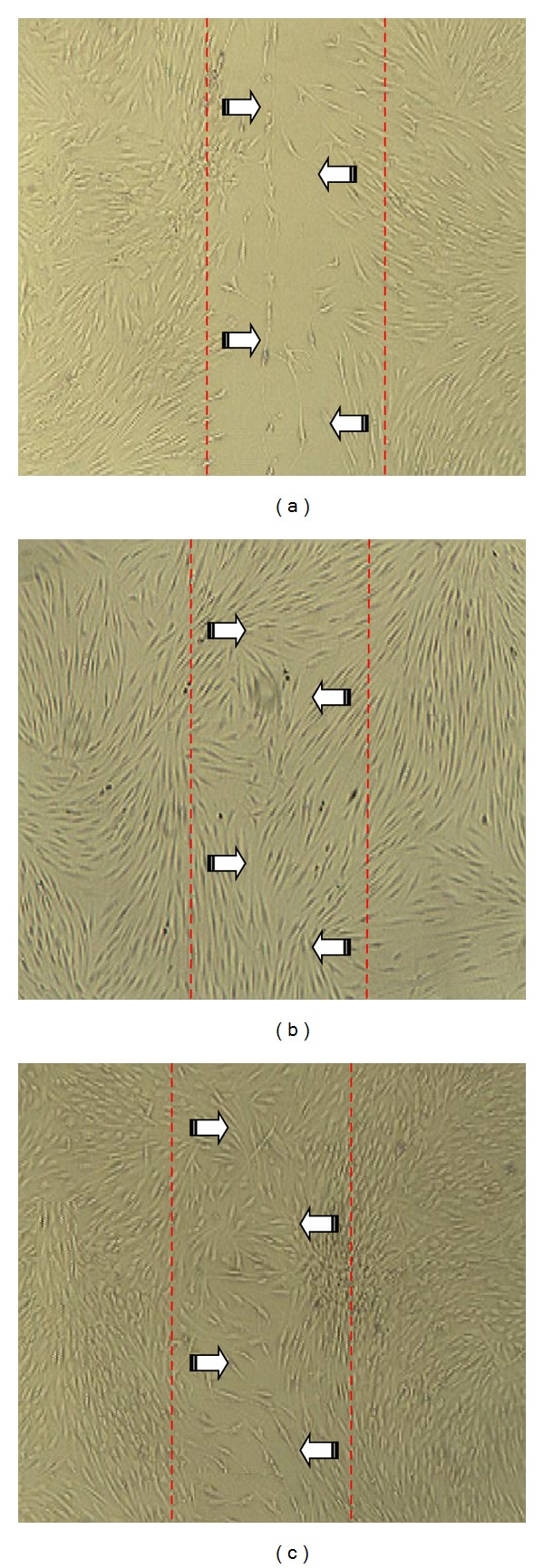
Micrographs showing the coverage of scratched wounds by HGF under various conditions at 42 h of incubation. (a) Negative control (HGF in basic media); (b) positive control (HGF treated with 10 ng/mL bFGF); and (c) test sample (HGF treated with 25 *μ*g/mL NSSE). The red lines marked the boundaries of the scratched wounds and the arrows indicated the direction of cells movement to cover the wound areas.

**Figure 3:**
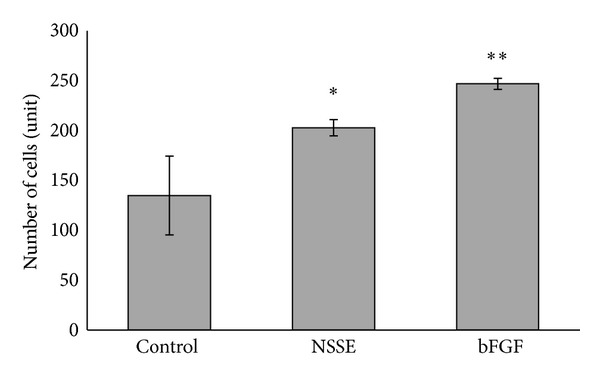
Quantitative measurement of cells number migrating in the corresponding scratched wound areas in Figure 2. The control was HGF in basic media, NSSE was the test sample at 25 *μ*g/mL, and bFGF was an enhancer added at 10 ng/mL. The values plotted were means of 3 determinations (*n* = 3). *P* values were indicated at *P* < 0.05 by (∗) and at *P* < 0.01 by (∗∗).

To ensure optimal activity of fibroblast is obtained in the wound closure assay; NSSE within the range of the EC_50_ was used as a working concentration in the assessment of wound healing activities.  Figure 2 exhibited the ability of NSSE-treated in comparison to bFGF-treated HGF to cover scratched wound areas made on the HGF cell monolayer. Compared to the negative control (Figure 2(a)), the enhancement of HGF proliferation and coverage of scratched wounds by NSSE (Figure 2(c)) were found to be significant but lower by about 33% comparable to the enhancement exhibited by growth factor bFGF (Figure 2(b)). This was quantitatively shown at 42 h of incubation period of HGF in the respective presence of NSSE and bFGF. 50% and 83% increase in migrated cell counts covering the wound areas were recorded in the presence of NSSE and bFGF, respectively (*P* < 0.05) (Figure 3).

**Figure 4:**
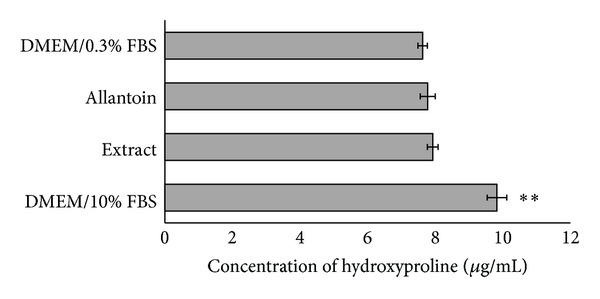
A bar chart indicating the production of collagen by HGF under various culture conditions. DMEM/0.3% FBS was a basic medium used as a negative control; DMEM/10% FBS was an enriched medium used as a positive control; allantoin (25 *µ*g/mL) is a skin enhancer often used in skincare products which was used for comparative purpose; and NSSE (25 *µ*g/mL) was the test sample. The values plotted were means of 6 determinations (*n* = 6). *P* values at *P* < 0.01 were indicated by (∗∗).

**Figure 5:**
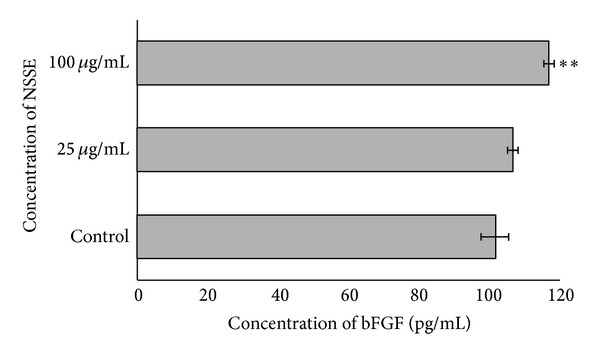
A bar chart showing the effect of NSSE on the production of bFGF by HGF. Two different concentrations at 25 and 100 *μ*g/mL were used and compared to a control. The values plotted were means of 3 determinations (*n* = 3). *P* values at *P* < 0.01 were indicated by (∗∗).

Based on the hydroxyproline assay, no significant difference in the level of hydroxyproline was observed between the basic medium (negative control), NSSE, and allantoin (Figure 4). This indicated that neither NSSE nor allantoin possessed the ability to enhance the production of collagen by HGF. The enriched medium (10% FBS) showed a significant 29% production of collagen compared to the basic medium (*P* < 0.05). NSSE was also found not to be very effective in enhancing the production of bFGF. Based on a quantitative sandwich immunoassay, only about 5% of bFGF increase was obtained following treatment of HGF with NSSE at the EC_50_ concentration. A higher concentration of NSSE (4-fold) was found to show better bFGF production of 15% (Figure 5).

**Figure 6:**
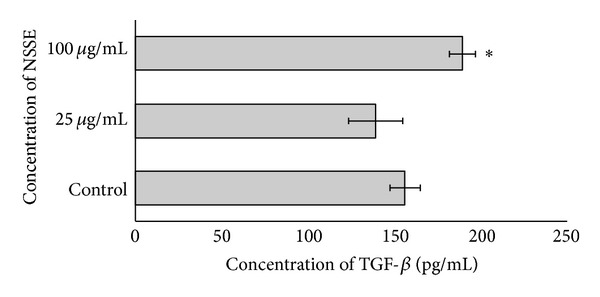
A bar chart showing the effect of NSSE on the production of TGF-*β* by HGF. Two different concentrations at 25 and 100 *μ*g/mL were used and compared to a control. The values plotted were means of 3 determinations (*n* = 3). *P* values at *P* < 0.05 were indicated by (∗).

With regard to the expression of TGF-*β*, it was observed that the concentration of TGF-*β* produced was 138.0 ± 15.6 pg/mL following treatment with 25 *μ*g/mL of NSSE and the production was increased to 188.0 ± 7.5 pg/mL with 100 *μ*g/mL of NSSE. From the graph shown in Figure 6, it was indicated that the production of TGF-*β* was not significantly decreased by the addition of 25 *µ*g/mL of NSSE (*P* > 0.05). Production of this growth factor, however, showed significant increase when treated with higher concentration of 100 *µ*g/mL of NSSE (*P* < 0.05).

## 4. Discussion

Evaluation and quantification of the biological activities of natural compounds are necessary before they are being offered to the market for human consumption. Besides exhibiting potentially beneficial therapeutic activities, other properties such as the toxic effect need to be recorded and thus require further characterization [27].

The experiments carried out in this study were focused at assessing the effects of aqueous extract of four medicinal plants in promoting the activities of wound healing, more specifically during the proliferative and remodelling phases of soft oral tissue healing. The proliferative phase of wound healing is characterized by the granulation of tissue formed mainly by the fibroblasts. During wound healing, along with angiogenesis, the reformulation and improvement of components of the collagen fiber are important to increase the tensile strength of the healing tissue [28]. The fast healing of wounds involving the oral mucosa has been associated with the expression of the extracellular matrix components such as procollagen I and tenascin C [5]. In this study, fibroblasts developed from an explant obtained from human gingival tissue were used to better represent cells of the human oral mucosa. Fibroblast cell cultures have been proposed to be a suitable method for testing wound healing activity* in vitro* [29].

It was found in this study that, among the four plants tested for wound healing activities of HGF, only NSSE showed significant positive and promising effects. At 25 *µ*g/mL, NSSE was able to enhance the proliferation of HGF by more than 50% (*P* < 0.05). However, the proliferative activity showed reduction at concentrations higher than 50 *μ*g/mL (Figure 1). Many reasons could have contributed to this effect and as reported in many studies involving natural compounds, this may possibly be due to increased presence of toxic components in the extract as the concentration is increased [30]. Hence, this explains the importance of IC_50_ determination and working within this range of concentration in studies involving natural products. Based on component profiling using LCMS, NSSE was found to contain polyphenols and flavonoids glycosides (unpublished results).

The strong proliferative activity of NSSE was reflected in the ability of the cells to increase in cell counts to cover the scratched wound areas (Figure 2). Scratch assay has been proven as a valuable and inexpensive tool to obtain first insights into how plant preparations or their isolated compounds can positively influence formation of new tissue [31]. The population of cells migrating into the scratched area treated with NSSE was enumerated 50% higher than that in the negative control (Figure 3). Although lower than the 83% cells increment determined in the presence of bFGF, this ability deemed the wound healing activity of NSSE to be further investigated. Growth factor bFGF was used in this study as a control because its significant fibroblast proliferative activity has been previously reported [32].

Collagen synthesis is essential for wound healing because, during the process, fibroblasts migrate towards the injured area and produce collagen to increase tissue permeability [24]. In this study, the hydroxyproline assay was carried out to determine the production of collagen by HGF. Hydroxyproline is an amino acid essential for collagen synthesis. For this reason, hydroxyproline content has been used as an indicator to determine the content of collagen [33]. Based on the results obtained, the synthesis of collagen by HGF was found not much affected by NSSE (Figure 4). Similar effect was also observed for allantoin although this white, nontoxic powder has been reported to promote epithelial stimulation [34] and used as a common agent in skincare products. One possible reason to explain for the low epithelial promoting activity of allantoin may be due to the different properties of the gingival cells (HGF) used in this experiment compared to epithelium of the skin that show histological as well as physiological differences.

The synthesis of extracellular matrix by fibroblasts during cellular proliferation involved intricate interactions between various growth factors and proteases [35]. Variations in the rate of cell proliferation have been associated with the differing expression of these growth factors. Two of the common and identified as key growth factors associated with wound healing are the basic fibroblast growth factor (bFGF) and transforming growth factor-*β* (TGF-*β*) [36, 37]. The former is one of the most potent stimulators of angiogenesis. bFGF is also mitogenic and chemotactic for both fibroblast and endothelial cells. The function of TGF-*β* on the other hand is believed to stimulate the synthesis of collagen and fibronectin [38]. TGF-*β* enhances mitogenesis of fibroblasts and smooth muscle cells but at the same time has also been shown to inhibit the mitogenic action of bFGF on endothelial cells.

NSSE was found to exhibit some effect on the level of growth factor bFGF and this effect was observed to be dose-dependent. The extract was able to enhance the expression of bFGF by 5% at 25 *μ*g/mL and 15% at 100 *μ*g/mL of extract, respectively (Figure 5). Although the influence of the extract at the lower concentration was not significantly different from the control, the effect shown at higher concentration though minor was significant (*P* < 0.01). The increased concentration of bFGF may be suggested to be a factor that had contributed to the fibroblast stimulating activity of NSSE. The increased level of bFGF by NSSE-treated HGF in a way supported the claim made by Hattori et al. that the growth factor bFGF is involved in gingival stimulation or gingival overgrowth.

Comparative to the effect of NSSE on the level of bFGF, slightly stronger influence of the extract on the expression of TGF-*β* was observed. Although a slight 12% reduction in TGF-B level was observed following the addition of 25 *µ*g/mL of the extract, this effect was not significantly different from that of the control. A more significant effect was determined at 100 *µ*g/mL whereby an increased expression of about 20% was recorded (Figure 6) to be higher than the 15% increase shown on bFGF (*P* < 0.05). However, reports and data on the expression of TGF-*β* by human gingival fibroblasts (HGF) are currently scarce to enable comparative analysis of the results obtained and thus require further investigations. Although the mechanism of wound healing would be expected to be similar in various tissues, the fact that wounds in the oral cavity often recovered in much shorter period deems for more tests to elucidate. The healing of wounds in the mouth is also expected to show some variations considering the different histological and anatomical features of both epithelia. In addition, the oral mucosa lining the surfaces of the mouth is kept moist by the presence of saliva and this different ecosystem within the oral cavity may influence the healing responses of an active agent.

It is thus suggested that, despite its low antioxidant property, the aqueous extracts of* N. sativa* seeds possess wound healing activities. This was based on the ability of NSSE to enhance the proliferation of fibroblasts and promote the level of bFGF. Although it was found to have no effect of collagen synthesis, NSSE accelerates wound closure activity. Based on these properties, NSSE has potential to be promoted as an agent for wound healing intended for use in the oral cavity. However, more studies are needed to provide supporting data.

## Figures and Tables

**Table 1:** DPPH scavenging activity of all four extracts in comparison to ascorbic acid. The values were the means ± SD of IC_50_ value. The experiment was carried out in triplicate with three determinations (*n* = 9).

Number	Samples	IC_50_ mean ± SD, (*μ*g/mL)
1	Control (ascorbic acid)	5.17 ± 1.07
2	*Nigella sativa* (*NSSE*)	961.0 ± 20.13
3	*Piper sarmentosum *	372.0 ± 8.19
4	*Pluchea indica *	143.0 ± 7.64
5	*Melastoma malabathricum *	353.0 ± 10.82
